# Zinc oxide nanoparticles mediated substantial physiological and molecular changes in tomato

**DOI:** 10.1371/journal.pone.0248778

**Published:** 2021-03-22

**Authors:** Fatemeh Pejam, Zahra Oraghi Ardebili, Alireza Ladan-Moghadam, Elham Danaee

**Affiliations:** 1 Department of Biology, Garmsar Branch, Islamic Azad University, Garmsar, Iran; 2 Department of Horticulture, Garmsar Branch, Islamic Azad University, Garmsar, Iran; National University of Kaohsiung, TAIWAN

## Abstract

There has long been debate about how nanoproducts meet agricultural requirements. This study aimed to investigate tomato responses to the long-time foliar application of zinc oxide nanoparticles (ZnO-NP; 0 and 3 mgl^-1^) or bulk type (BZnO). Both ZnO-NP and BZnO treatments, especially the nanoform, were significantly capable of improving growth, biomass, and yield. The ZnO-NP treatment upregulated the expression of the *R2R3MYB* transcription factor by 2.6 folds. The BZnO and ZnO-NP treatments transcriptionally up-regulated *WRKY1* gene by 2.5 and 6.4 folds, respectively. The *bHLH* gene was also upregulated in response to BZnO (2.3-fold) or ZnO-NP (4.7-fold). Moreover, the ZnO-NP application made a contribution to upregulation in the *EREB* gene whereas the bulk compound did not make a significant change. Upregulation in the *HsfA1a* gene also resulted from the ZnO-NP (2.8-fold) or BZnO (1.6-fold) supplementation. The *MKK2* and *CAT* genes displayed a similar upregulation trend in response to the supplements by an average of 3-folds. While the application of ZnO-NP slightly down-regulated the histone deacetylases (*HDA3*) gene by 1.9-fold, indicating epigenetic modification. The supplements, especially the nano-product, enhanced concentrations of K, Fe, and Zn in both leaves and fruits. The concentrations of Chla, Chlb, and carotenoids were increased in response to the BZnO or ZnO-NP treatments. Likewise, BZnO or ZnO-NP mediated an increase in activity of nitrate reductase and proline content in leaves. These treatments increased soluble phenols and phenylalanine ammonia-lyase activity. With a similar trend, the BZnO or ZnO-NP application improved the activities of catalase and peroxidase enzymes. The reinforcement in metaxylem and secondary tissues resulted from the applied supplements. This study provides comprehensive comparative evidence on how ZnO-NPs may remodel the chromatin ultrastructure and transcription program, and confer stress tolerance in crops. This study also underlines the necessity of providing integrated transcriptome and proteome data in future studies.

## Introduction

Nowadays, nanotechnology provides great opportunities for development in a plethora of disciplines, including biotechnology and agriculture [1–3]. Taking account of agriculture’s requirements, attempts have been made to enhance crop productivity and fruit quality, to improve immunity, and to reduce the consumption of chemical fertilizers/pesticides [4,5]. In this regard, nano-based products have exhibited a great potency to increase crop production efficiency [6,7] and to improve plant tolerance to abiotic and biotic stresses [8,9] in crops. However, there has been a widespread debate surrounding the potential benefits and phytotoxicity associated with the application of nanomaterials. Among the most widely applied nanoproducts, investigation of synthesis (physical, chemical, and biological methods) and potential benefits/risks of metal oxides are gaining great attention due to their unique characteristics [10,11]. Both Zinc oxide (ZnO) and its nanoparticles (ZnO-NPs) are widely functionalized in a plethora of industrial activities [12]. Some studies have highlighted the potential functions of ZnO-NPs to decontaminate seeds [13], increase biomass [12–15], enhance nutrition, especially Zn bioaccumulation [12,13,15], up-regulate primary metabolism [3,12,13], stimulate secondary metabolism [12,13], and improve yield [16]. ZnO-NPs at optimal concentration, additionally, conferred tolerance to stress conditions, like a drought in sorghum [17], Cd in wheat [18], Cd in tomato [6], and Cd in maize [19]. However, there is inadequate knowledge of the molecular mechanisms involved in plant interaction with ZnO-NPs [20].

Transcription factors and mitogen-activated protein kinases (MAPKs) are regulatory proteins that contributed to gene regulation and signal transduction [21,22]. We, therefore, attempted to monitor the ZnO-NP-mediated transcriptional variation in several important transcription factors, including *R2R3MYB* [23], *bHLH* [24], *WRKY1* [21], *EREB* [9], *HsfA1a* [25], and *MKK2* [26]. These proteins play fundamental regulatory roles in the expression of a wide spectrum of downstream genes and act as integrating components of diverse signaling pathways, thereby modulating growth program, tissue differentiation, primary and secondary metabolism, metabolism and signaling of hormones, and defense responses [9,21,23–26].

Our next aim was to explore the epigenetic response to ZnO-NPs. Recently, it has been hypothesized that nanoparticles can be associated with epigenetic responses, like cytosine DNA methylation [27,28] and histone modification [12]. Histone deacetylases (HDA) mediate the process by which chromatin ultrastructure can be remodeled from euchromatin to heterochromatin. This is an epigenetic modification that determines gene accessibility to transcriptional proteins. Taking account of knowledge gaps on the epigenetic responses of plants to ZnO-NPs, the *HDA3* gene was among our target genes.

Nearly most previous studies on ZnO-NPs have been investigated plant responses to short-time application methods (especially at high concentrations) at early developmental stages. However, it remains unknown how plants may respond to long-time exposure to ZnO-NPs at low concentrations. We aimed to investigate the plant responses to the foliar application of ZnO-NPs or its bulk counterpart at various aspects, including [I] biomass and yield, [II] expression of above-mentioned transcription factors, [III] epigenetic response according to the transcriptional change in *HDA3* gene, [IV] expression of *CAT* as a target gene in antioxidant system, [V] expression of *MKK2*, [VI] nutritional status, [VII] biochemical traits, and [VIII] stem anatomy.

## Material and methods

### Treatments and experimental conditions

Tomato seeds (KARNAK F1) were supplied by Fito company, India imported by BAZRGANKALA company. The ZnO-NP product with sizes ranging from ten to thirty nm (US research nanomaterials, Inc; 3302 Twig Leaf Lane Houston, TX 77084, USA) was supplied for this experiment. The UV-visible spectrum of ZnONP displayed a peak in the UV region. According to the zeta potential index, ZnONPs possess an appropriate amount of -20 mV that is acceptable for suitable electrostatic repulsion and stability.

This experiment was conducted under soilless conditions (pots containing Cocopeat and Perlite at a ratio of 70: 30). The irrigation of tomato plants was performed using a Hoagland nutrient solution 3 times a week and water for the rest. One-month-old seedlings were sprayed with ZnO-NP at two concentrations (0 and 3 mgl^-1^) or corresponding concentrations of its bulk compound (BZnO; 15 times with 72 h intervals for two months). Harvesting of plants was performed at 2 stages; Six pots in each treatment group were harvested in the first stage (one week after the last sprays for evaluating the growth, anatomical, biochemical, and molecular analysis) and three pots in each treatment group were harvested to evaluate the crop yield in the second stage.

### RNA extraction and real-time quantitative PCR (qRT-PCR)

Trizol (GeneAll Biotechnology Co, South Korea), DEPC Water (Bio Basic, Canada), RNA isolation kit (Denazist, Iran), and Dnase I (Thermo Fisher, USA) were employed to prepare total RNA extract from the leaves. Then, the absorbance ratio (260/280 nm) of RNA extract was monitored by the Nanodrop instrument (Thermo Scientific™NanoDrop Model 2000c) to estimate RNA purity. After that, complementary DNA (cDNA) was generated using a thermocycler (PEQLAB, 96Grad). To design the primers of target genes, Oligo7 and AllelID software was utilized. The forward and reverse sequences of primers for target genes, including histone deacetylase (*HDA3*, NM_001365152.1), Catalase (*CAT1*; NM_001247898), mitogen-activated protein kinase *MAPKK* (MKK2; AF104247), ethylene-responsive element-binding protein (EREB; AF502085), *MYB* transcription factors (*R2R3MYB*; NM_001247333), *WRKY1* transcription factor (AK247980.1), basic/helix-loop-helix (*bHLH*; NM_001346305), heat stress transcription factor *HsfA1a* (*HsfA1a*; NM_001309248), and Elongation factor (a housekeeping gene) are presented in Table 1. Finally, the real-time quantitative PCR (Applied Biosystems StepOne™ Real-Time PCR) was conducted under a cycling program (94°C for 120s, 94°C for 15s, 57°C for 25s, and 72°C for the 20s) to investigate transcriptional variation between the treatment groups and control. To evaluate the transcription difference, the calculation of the relative fold expression of each gene was performed according to the delta-delta Ct method (2^-ΔΔCT^ formula).

**Table 1:** The forward (F) and reverse (R) sequences of primers for the target genes, including *EREB*, *WRKY1*, *HDA3*, *bHLH*, *R2R3MYB*, *HsfA1a*, *CAT*, and *MKK2*, and Elongation factor (a housekeeping gene).

Primer name	F/R	Sequence (5’-3’)	length	Tm	Amplicon (bp)
*EREB*	F	GAGAAGTCGTACCGAGGTGTT	21	60	119
*EREB*	R	GCATCTTCCGCGCTATCAAAT	21	60	
*WRKY1*	F	GTGAATCACGACACATTGTTCA	22	59	170
*WRKY1*	R	GGATGACCTCTCCACATGCTTC	22	60	
*HDA3*	F	GCACCGTATCAGAATGGCCC	20	61	96
*HDA3*	R	ATATCATCCTCTCCCGCCGG	20	61	
*EFa*	F	TTCACATCAGCATCGTGGTCA	20	61	127
*EFa*	R	TCAGCAGCTTCCTTCTCGAAC	21	60	
*bHLH*	F	TCCGCCTTTCTAGGGTTCTTC	21	60	134
*bHLH*	R	CCTTGGCTGATCAGTACTGGA	21	60	
*R2R3MYB*	F	TGCACCTAACTCACCACAACT	21	60	115
*R2R3MYB*	R	CTCCGACGATGATGACTCGAT	21	60	
*HsfA1a*	F	AGTCGACGTAAACCTGCTCAT	21	60	129
*HsfA1a*	R	CCCAAATTTCCCAACCTCGAC	21	60	
*CAT1*	F	AACAACTCGCGTTTAACCCTG	21	60	110
*CAT1*	R	TGGTCCAATACGGTGTCTCTG	21	60	
*MKK2*	F	CTGTTCCTCTCCCTCTTCCAC	21	60	107
*MKK2*	R	GCCACTACCGATGCGATTAAC	21	60	

### Nitrate reductase, peroxidase, catalase, and phenylalanine ammonia-lyase (PAL)

In this study, the BZnO- or ZnO-NP-mediated changes in several important enzymes involved in nitrogen primary metabolism (nitrate reductase), enzymatic antioxidants (catalase and peroxidase), and secondary metabolism (PAL) were investigated. To extract enzymes from leaves, the phosphate buffer (100 mM; pH of 7.2) was utilized. After that, the resulting homogenates were centrifugated at 4°C and the supernatants were kept as enzyme extract at −80°C. The nitrate reductase activity was determined according to the procedure of Sym [29] and expressed in terms of micromole nitrite per hour per gram fresh weight. The activities of enzymatic antioxidants, including catalase [30] and peroxidase [30] were quantified and expressed in terms of unit enzyme per gram fresh mass. Additionally, the PAL activity was estimated based on the conversion rate of phenylalanine amino acid to cinnamic acid and calculated in terms of microgram cinnamate per hour per gram fresh mass [31].

### Photosynthetic pigments, proline, and soluble phenols

To determine the BZnO- or ZnO-NP-associated variation in photosynthesis, acetone solvent was applied to purify photosynthetic pigments, including chlorophyll a (Chla), chlorophyll b (Chlb), and carotenoids from leaves. Absorption rates of the resulting extracts were spectrophotometrically measured at different wavelengths, including 470, 663, and 646 nm. After that, the concentrations of Chla, Chlb, and carotenoids were quantified using the equations represented by Lichtenthaler and Welburn [32]. Moreover, the procedure of Bates et al., [33] was applied to measure the concentration of proline amino acid in leaves. Total soluble phenols were extracted using ethanolic solvent and then the phenol content was spectrophotometrically estimated according to the Folin-Ciocalteu assay [30].

### Measurement of K, Zn, and Fe in both leaves and fruits

To measure K, Zn, and Fe contents, dry ashes of both leaves and fruits were prepared using a thermal decomposition procedure in a furnace (680°C). Next, a solvent mixture consisting of nitric acid and hydrochloric acid (2:1) was applied to dissolve the resulting ash. After that, K content was assayed by a flame photometric protocol (Flame photometer, JENWAY). Besides, Fe and Zn levels were determined using the Atomic Absorption Spectroscopy assay (Atomic Absorption Spectrophotometer; VARIAN; AA240).

### Histological studies

To study the potential anatomical responses, we prepared the hand-made cross-section of the stem organ (50 cm from the stem apical meristem). After that, these sections were subject to preparing and staining steps, including Sodium hypochlorite for 30 min, 3% acetic acid for 5 min, soaking in Zaji carmen stain for 25 min, and exposure to methylene blue for 40 s; each step followed by washing with distilled water [27]. Finally, the microscopical images of stained stem cross-sections were recorded.

### Statistical analysis

The experimental design was completely randomized. GraphPad software was applied to perform an analysis of variance (ANOVA) on data. The differences among the mean values of treatment groups were statistically compared according to the Tukey test analysis.

## Results

**Fig 1:**
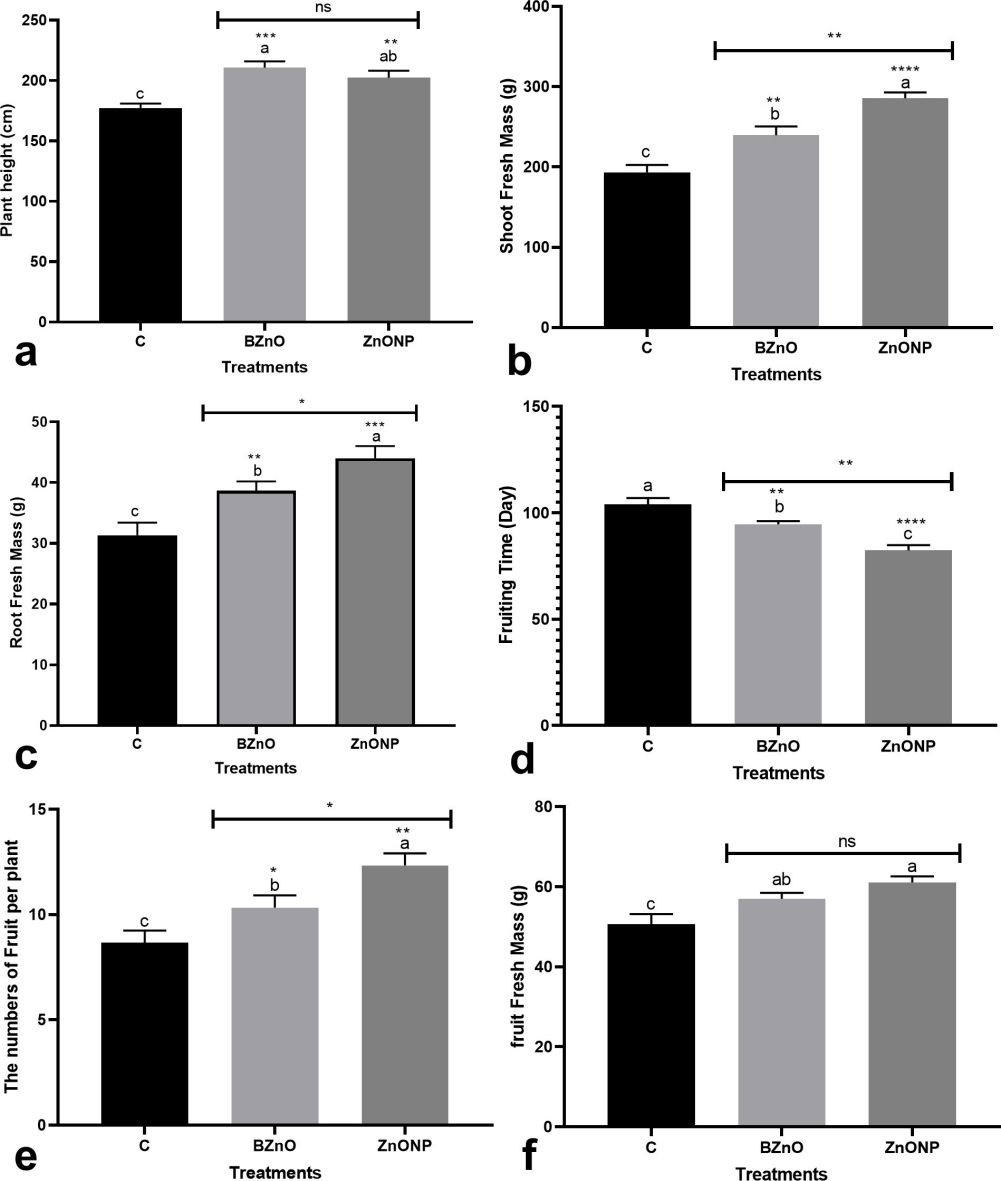
The BZnO- and ZnO-NP-mediated changes in the growth and yield-related characteristics, including plant height [a], shoot fresh mass [b], root fresh mass [c], fruiting time [d], the numbers of fruit per plant [e], and fruit fresh mass [f]. Different letters on top of columns refer to statistically significant differences according to Tukey’s multiple comparisons test. ns: non-significant; *: 0.01<p≤0.05; **: 0.001<p≤0.01; ***: 0.0001<p≤0.001; ****: p≤0.0001. The p-value levels for comparing the mean of each group vs the control group are displayed by the asterisk on each column. Also, an asterisk [*] on the drawn lines presents the p-value levels for comparison between the BZnO and ZnONP groups.

Both ZnO-NP and BZnO significantly increased shoot height when compared to the control (Fig 1A). The ZnO-NP treatment significantly enhanced shoot biomass by 47.8% while the BZnO application increased this trait by 23.8% (Fig 1B). With a similar trend, both supplements significantly augmented root fresh mass by an average of 32% (Fig 1C). The difference in shoot and root biomass between the BZnO and ZnO-NP groups was statistically significant. The ZnO-NP and BZnO treatments, especially the nanoform, accelerated entry into the reproductive phase by an average of 15% relative to the control (Fig 1D). The applied supplements, especially the nano-compound, increased the crop yield in terms of the number of fruits (Fig 1E) and the fruit fresh mass (Fig 1F).

**Fig 2:**
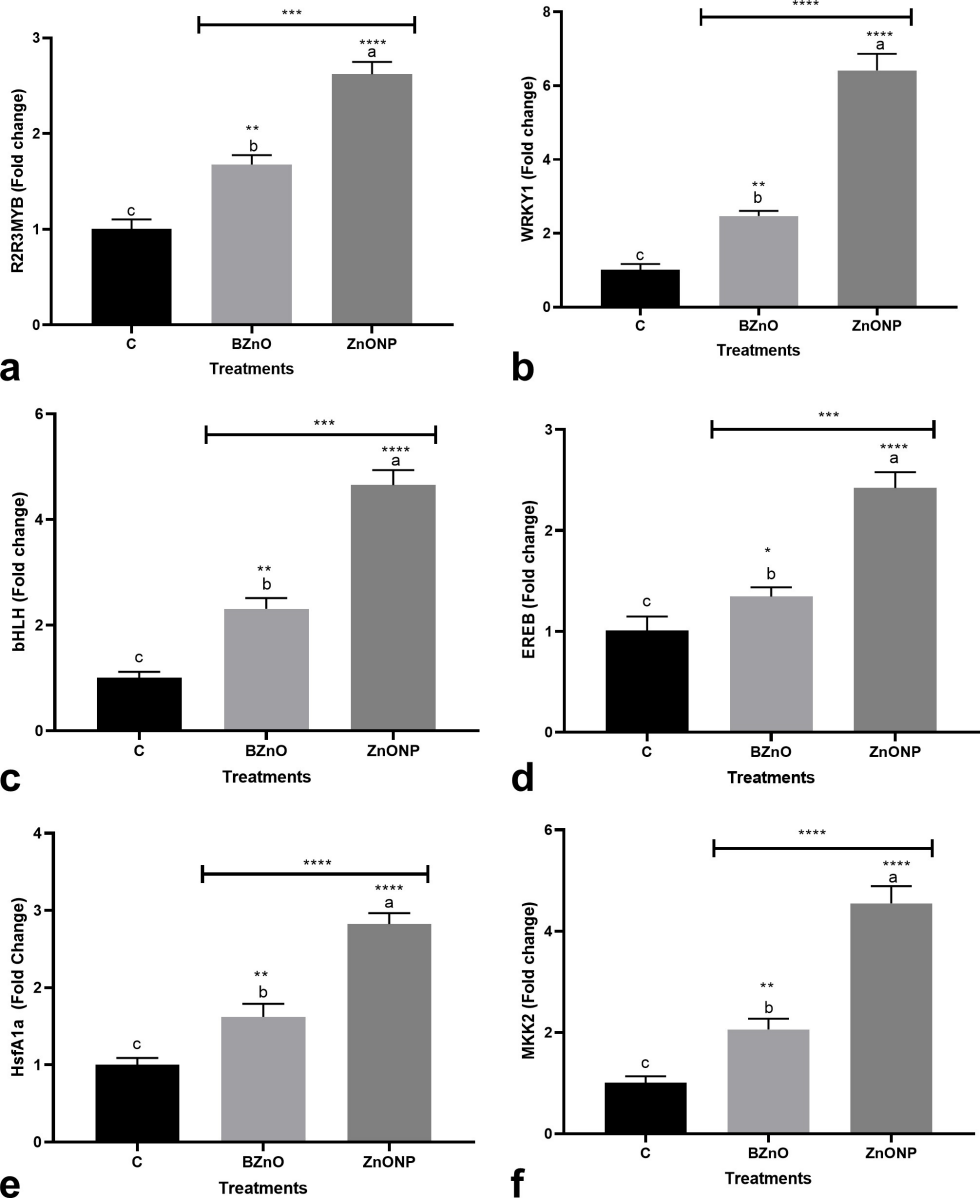
The variations in the expression of several transcription factor genes, including *R2R3MYB* [a], *WRKY1* [b], *bHLH* [c], *EREB* [d], and *HsfA1a* [e] and *MKK2* [f] as molecular responses to the long-time foliar application of BZnO and ZnO-NP at a low dose. Different letters on top of columns refer to statistically significant differences according to Tukey’s multiple comparisons test. ns: non-significant; *: 0.01<p≤0.05; **: 0.001<p≤0.01; ***: 0.0001<p≤0.001; ****: p≤0.0001. The p-value levels for comparing the mean of each group vs the control group are displayed by the asterisk on each column. Also, an asterisk [*] on the drawn lines presents the p-value levels for comparison between the BZnO and ZnONP groups.

**Fig 3:**
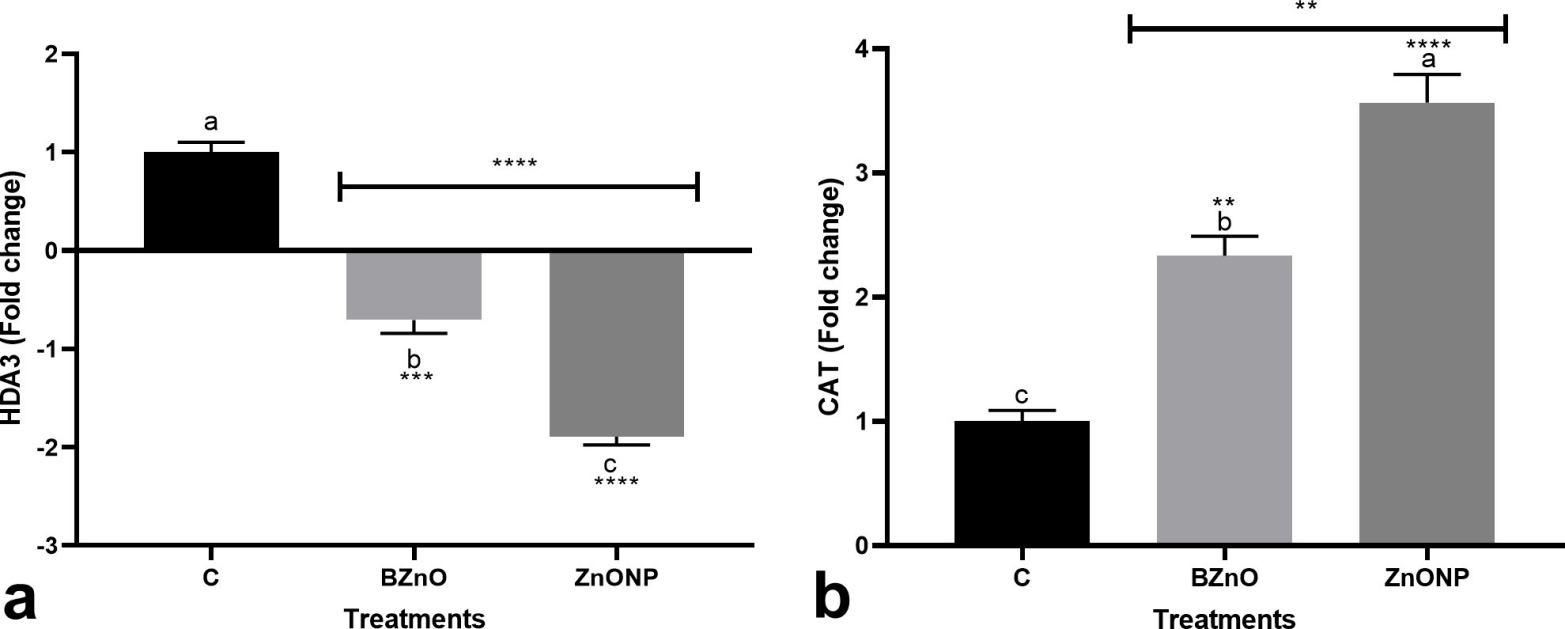
The variation in the expression of *HDA3* [a] and *CAT* [b] in response to the long-time foliar application of BZnO and ZnO-NP; Different letters on top of columns refer to statistically significant differences according to Tukey’s multiple comparisons test. ns: non-significant; *: 0.01<p≤0.05; **: 0.001<p≤0.01; ***: 0.0001<p≤0.001; ****: p≤0.0001. The p-value levels for comparing the mean of each group vs the control group are displayed by the asterisk on each column. Also, an asterisk [*] on the drawn lines presents the p-value levels for comparison between the BZnO and ZnONP groups.

The ZnO-NP group (2.6-fold) displayed a significantly higher expression of the *R2R3MYB* transcription factor when compared to the BZnO and control groups (Fig 2A). The BZnO and ZnO-NP treatments significantly up-regulated *WRKY1* by 2.5 and 6.4 folds, respectively, over the control (Fig 2B). The BZnO treatment slightly upregulated the *bHLH* gene by 2.3-fold (Fig 2C). While the ZnO-NP treatment moderately stimulated the transcription of the *bHLH* gene by 4.7 folds over the control (Fig 2C). The *EREB* gene was significantly up-regulated in response to the ZnO-NP treatment. However, the foliar utilization of BZnO did not make a significant change in the expression of the *EREB* gene (Fig 2D). The significant upregulation in the HsfA1a gene resulted from the ZnO-NP (2.8-fold) or BZnO (1.6-fold) supplementation (Fig 2E). The BZnO- or ZnO-NP-treated seedlings displayed a significantly higher expression of the *MKK2* gene by 2.1 and 4.5 folds, respectively over the control (Fig 2F). The foliar application of ZnO-NP significantly down-regulated the *HDA3* gene by 1.9-fold when compared to the control (Fig 3A). The statistical analysis revealed that the BZnO and ZnO-NP treatments induced the *CAT* gene by 2.3 and 3.6 folds, respectively when compared to the control (Fig 3B).

**Fig 4:**
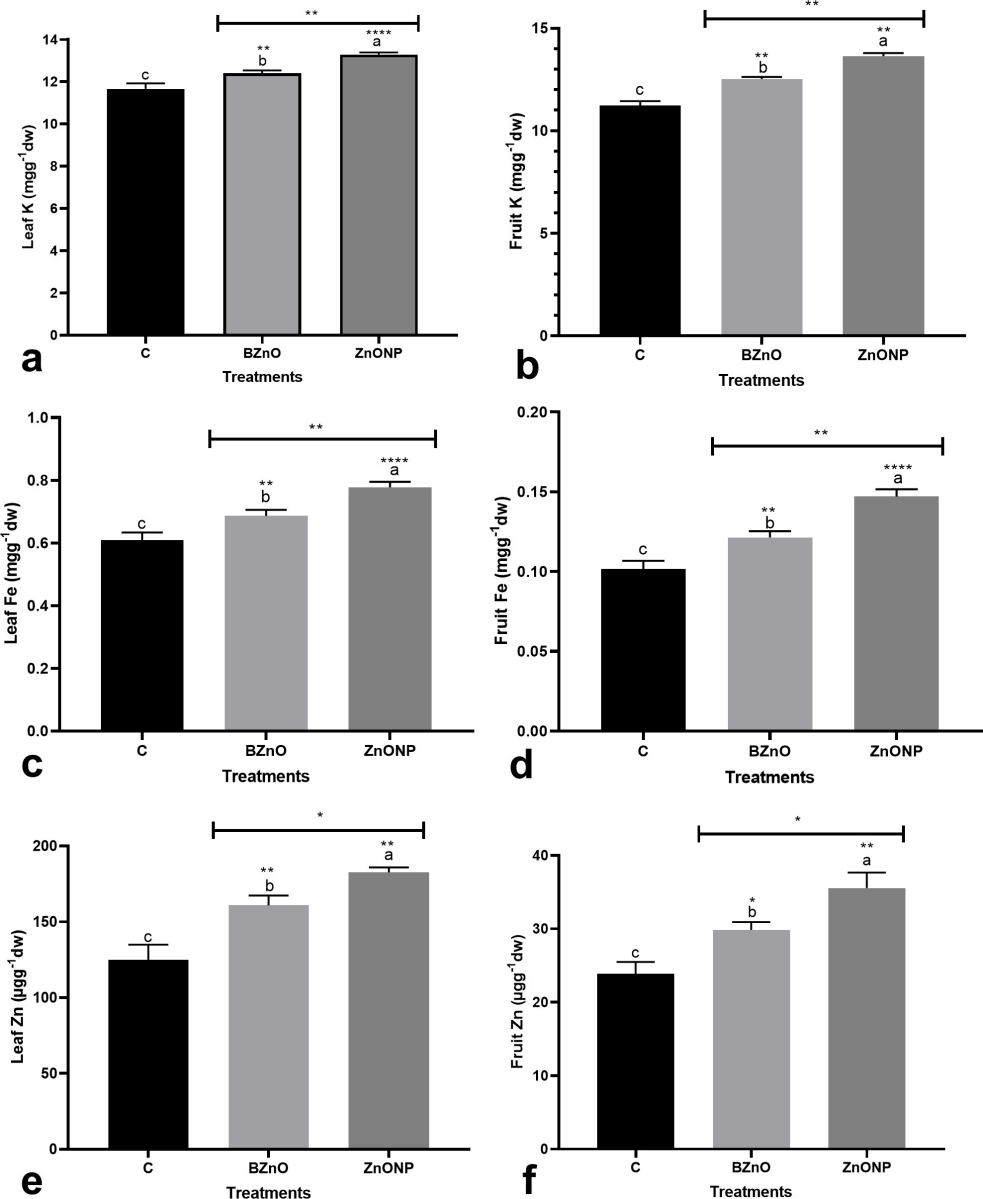
The modification in the concentration of leaf K [a], fruit K [b], leaf Fe [c], fruit Fe [d], leaf Zn [e], and fruit Zn [f] in response to the long-time foliar application of BZnO and ZnO-NP at a low dose. Different letters on top of columns refer to statistically significant differences according to Tukey’s multiple comparisons test. ns: non-significant; *: 0.01<p≤0.05; **: 0.001<p≤0.01; ***: 0.0001<p≤0.001; ****: p≤0.0001. The p-value levels for comparing the mean of each group vs the control group are displayed by the asterisk on each column. Also, an asterisk [*] on the drawn lines presents the p-value levels for comparison between the BZnO and ZnONP groups.

The ZnO-NP treatment slightly enhanced K concentration in leaves and fruits by 19.8% and 21%, respectively when compared to the control (Fig 4A and 4B). While the BZnO application increased K concentration in both leaves and fruits by 15.2% and 11.5%, respectively over the control. With a similar trend, the BZnO and ZnO-NP treatments mediated a significant increase in Fe concentration in leaves by 12.7% and 27.5%, respectively (Fig 4C and 4D). Similarly, the BZnO- and ZnO-NP-treated seedlings had a significantly higher concentration of Fe in fruits by an average of 31% over the control (Fig 4C and 4D). The efficiency of ZnO-NP in increasing the Zn content in both leaves and fruits was significantly higher than in bulk form (Fig 4E and 4F).

**Fig 5:**
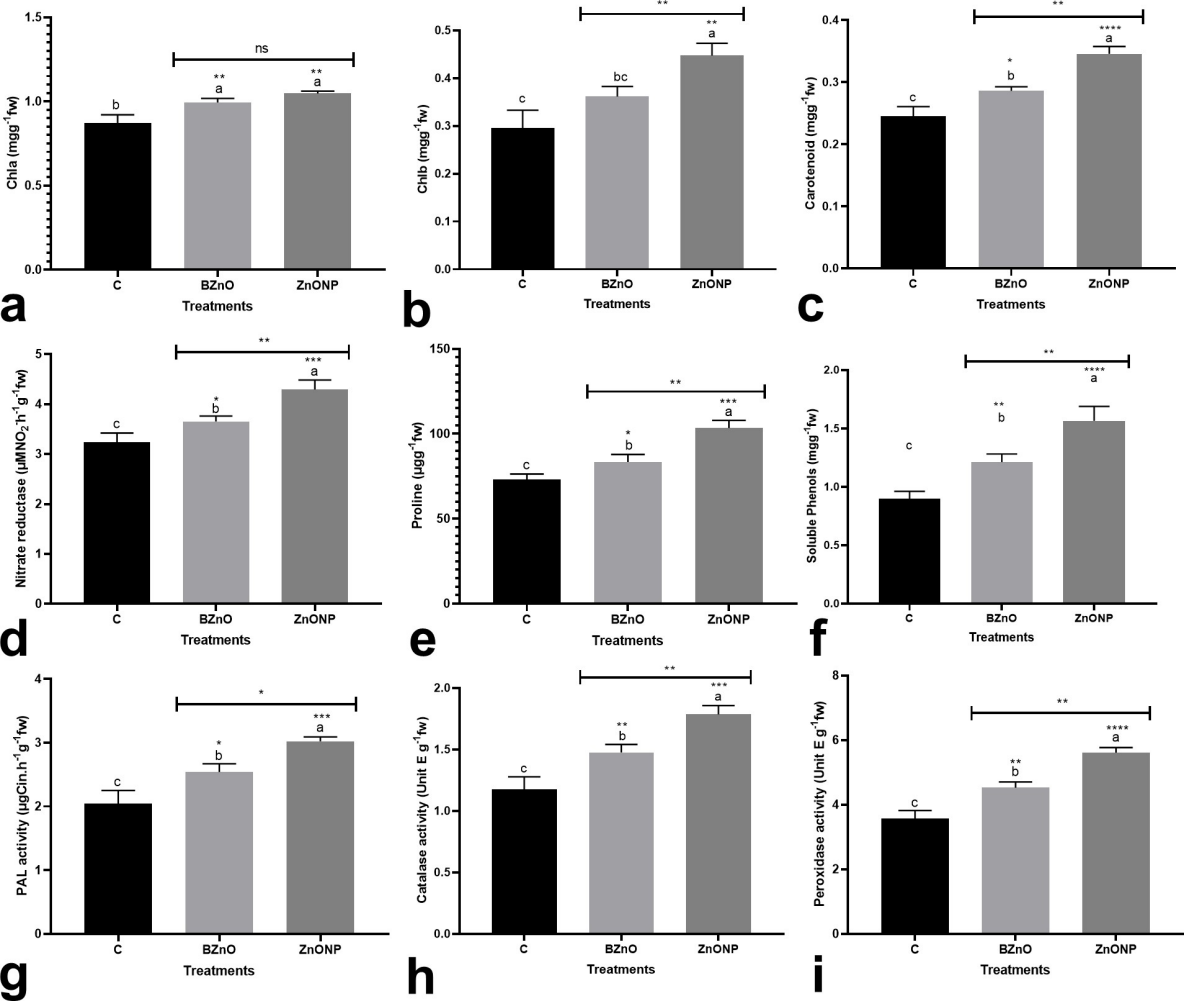
The BZnO and ZnONP-mediated variations in diverse important physiological traits, including the concentrations of Chla [a], Chlb [b], carotenoids [c], the activity of nitrate reductase enzyme [d], proline content [e] soluble phenols [f], PAL activity [g], catalase activity [h], and peroxidase activity [i]; Different letters on top of columns refer to statistically significant differences according to Tukey’s multiple comparisons test. ns: non-significant; *: 0.01<p≤0.05; **: 0.001<p≤0.01; ***: 0.0001<p≤0.001; ****: p≤0.0001. The p-value levels for comparing the mean of each group vs the control group are displayed by the asterisk on each column. Also, an asterisk [*] on the drawn lines presents the p-value levels for comparison between the BZnO and ZnONP groups.

The BZnO and/or ZnO-NP-treated seedlings contained a higher concentration of Chla by an average of 17.1% over the control (Fig 5A). Similarly, a significant increase in Chlb content by an average of 36% was observed in the BZnO and/or ZnO-NP-treated seedlings (Fig 5B). The concentration of carotenoids was increased in response to the BZnO or ZnO-NP treatment by an average of 28% when compared to the control (Fig 5C). Except for Chla, the difference between BZnO and ZnO-NP groups was statistically significant in terms of Chlb and carotenoids. The BZnO or ZnO-NP, especially the latter, mediated a significant increase in the activity of nitrate reductase by an average of 23% (Fig 5D). In this regard, the difference between the BZnO and ZnO-NP treatment groups was statistically significant. Likewise, the BZnO and ZnO-NP groups had a significantly higher concentration of proline (about 27%) in comparison to the control (Fig 5E). The BZnO and ZnO-NP treatments also resulted in a significantly higher concentration of soluble phenols by an average of 54.5% when compared to the control (Fig 5F). The PAL activity was significantly upregulated following the BZnO or ZnO-NP treatments by an average of 36% (Fig 5F). Similarly, the BZnO and ZnO-NP treatments significantly improved catalase activity by 25.5% and 51.7%, respectively, over the control (Fig 5H). Peroxidase activity also showed an increasing trend in response to the BZnO or ZnO-NP treatments by 26.9% and 57.3%, respectively relative to the control (Fig 5I).

**Fig 6:**
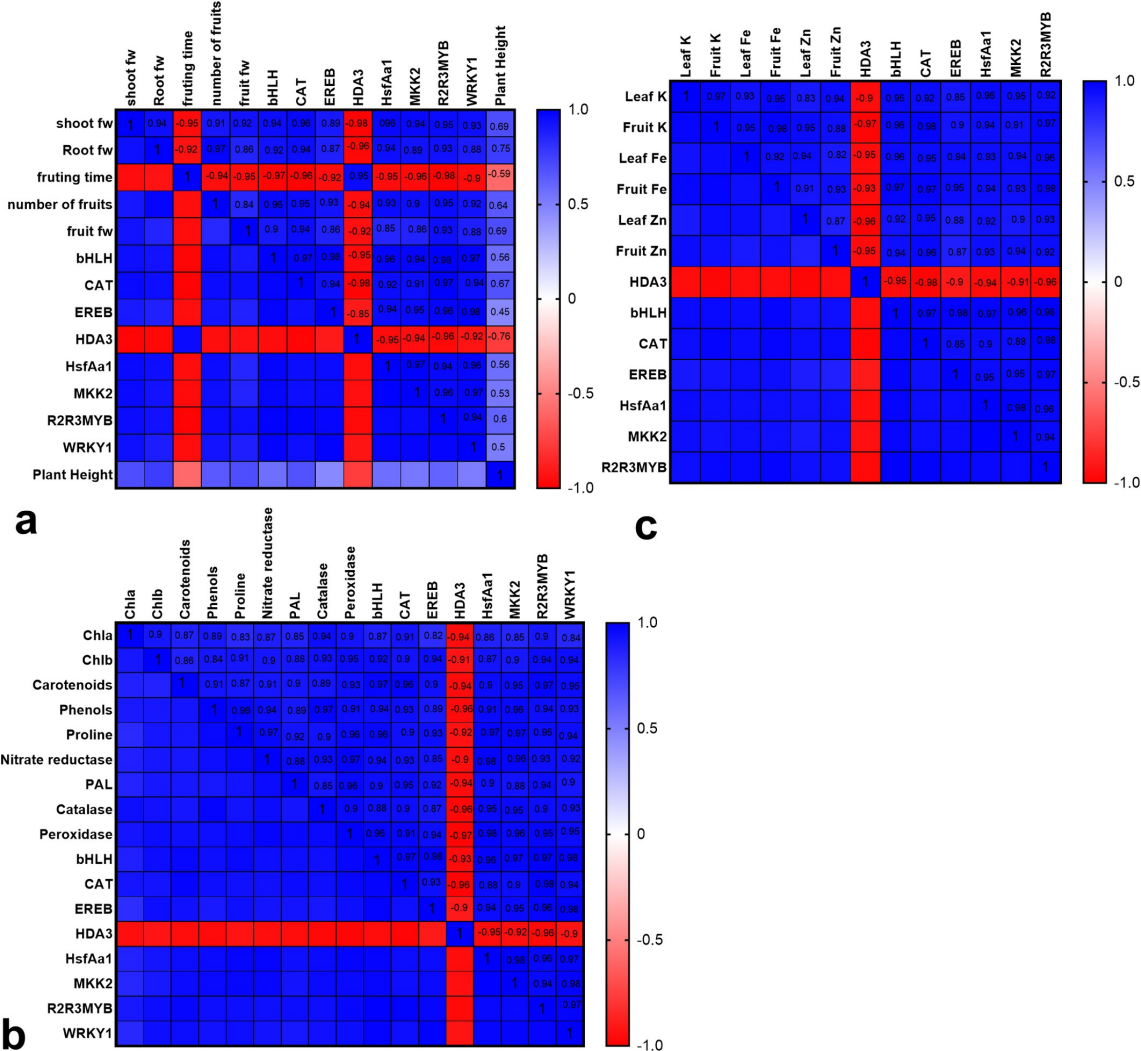
Heatmap correlation matrix among target genes and growth/yield-related traits [a]; Heatmap correlation matrix among target genes and several physiological characteristics [b]; Heatmap correlation matrix among target genes and several investigated nutrients [c].

The statistical analysis confirmed the strong correlation between each target gene and growth/yield-related traits (Fig 6A). Correlation among the target genes and the evaluated physiological characteristics, including photosynthetic pigments, nitrate reductase, proline concentration, defense-related enzymes (peroxidase, catalase, and PAL), concentrations of nutrients, etc. displayed a similar significant trend (Fig 6B and 6C).

**Fig 7:**
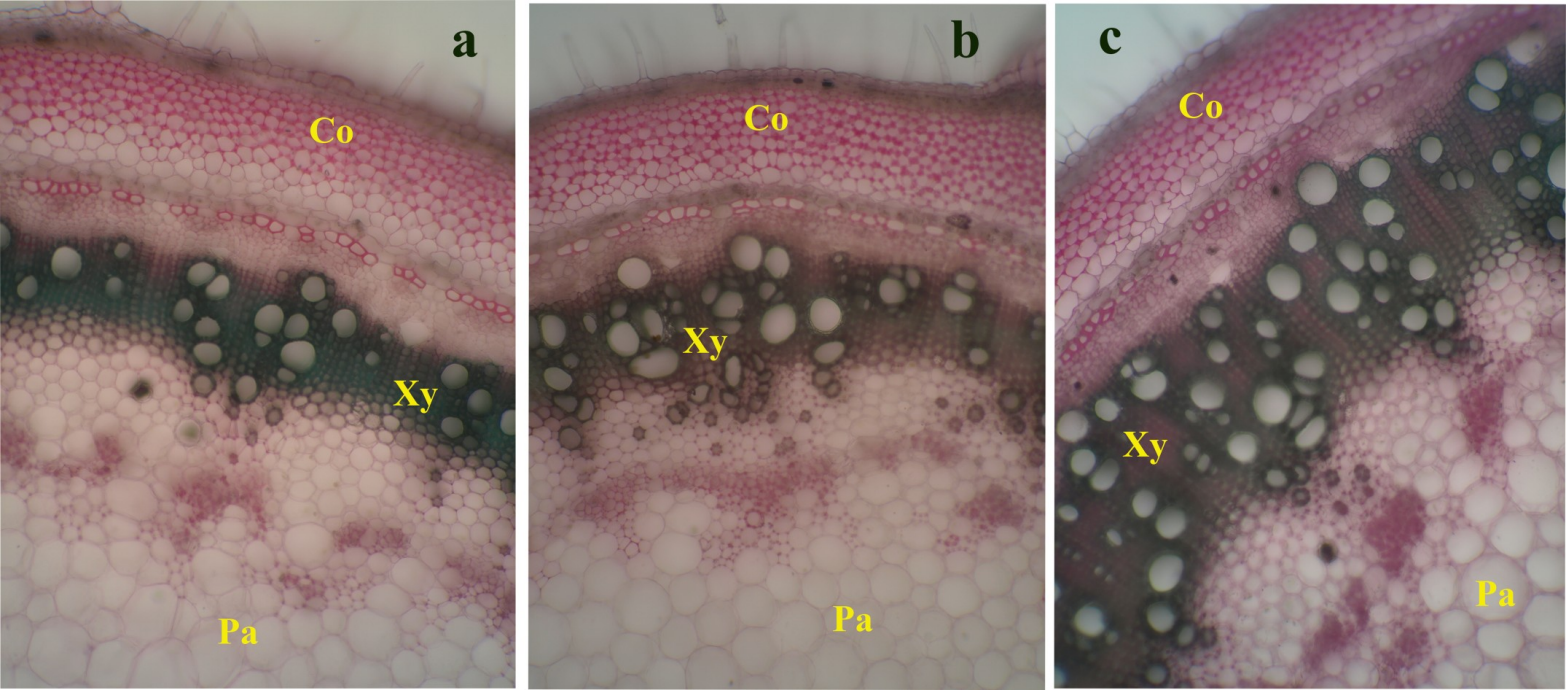
BZnO and ZnONP- associated anatomical variation in stem cross-sections at 50 cm distance from the stem apical meristem; Treatment groups: a- Control; b- BZnO group; c- ZnONP group; Abbreviations: Co- Collenchyma; Xy- Xylem tissue; Pa- Parenchyma.

Fig 7 displays the anatomical differences in the stem structure in response to ZnONP or its bulk. Both BZnO and ZnONP treatments modified the stem anatomy, especially in terms of metaxylem and fiber secondary tissues.

## Discussion

The long-time application of ZnONP or its bulk counterpart contributed to modifying vegetative growth, reproductive development, yield, nutritional status, nitrogen assimilation, photosynthesis, enzymatic antioxidant system, secondary metabolism, anatomy, and transcription of important genes. Zn acts as a significant signal and plays pivotal functions, like cofactor in a plethora of enzymes (notably in DNA-binding proteins), maintaining genetic stability, regulating DNA methylation, transcription, translation, hormone metabolism, cellular differentiation, and cell proliferation [20,34]. Variation in growth, metabolism, transcriptome, and proteome are, therefore, expected following Zn supplementation. In line with our results, several reports provide a clear illustration of ZnO-NP benefits in improving photosynthesis [15], nutrition [13], growth [14], secondary metabolism [12,13,35], antioxidant defense system [18], Zn bioaccumulation [36], fertility [16], and immunity [6,19,35]. However, researchers firmly believe that ZnO-NP at high doses is hazardous to crops [12,13]. This study provides evidence on how ZnO-NP is more capable of improving crop growth, yield, and metabolism and modifying transcriptional programs than the bulk form. Moreover, this study showed comprehensive comparative evidence in terms of physiological and molecular responses of tomato to ZnO-NP vs the bulk form, while a vast majority of previous reports have only reported the physiological responses of plants to nanoparticles, or the comparison data was limited to growth parameters. However, the mechanism through which ZnO-NP is capable of conferring partly differential responses remains controversial. A majority of researchers believe that differential physicochemical characteristics of nanoparticles relative to the bulk contributed to differences in their uptake kinetics, subsequent interactions with biomolecules, signaling, and responses [1–3,10–12,22,27,28]. More studies are, therefore, required to confirm this hypothesis.

ZnO-NP moderately up-regulated several target important genes, including *MKK2*, *bHLH*, *WRKY1*, *EREB*, *HsfA1a*, and *R2R3MYB*. These remodeling in the transcription profile of genes can be explained by ZnONP-associated alterations in signaling molecules [20,37] and hormones [13,20]. Statistical analysis confirmed the strong correlation among the growth/yield-related traits, the target transcription factors, and *MKK2*. Besides, correlations among these genes and investigated physiological traits displayed a similar trend. These findings highlight the fundamental involvement of *bHLH*, *EREB*, *WRKY1*, *HsfA1a*, and *R2R3MYB* transcription factors and *MKK2* in regulating plant growth and metabolism in response to ZnO-NP. Our results are consistent with the finding of Vafaee Moghadam et al. [12] who recently reported the transcriptional up-regulation in *bZIP*, *AREB*, and *WRKY1* transcription factors following treatment of *Datura stramonium* plants with ZnONP. HSFs [25], bHLH [38], and EREB [9,12] are fundamental regulatory proteins involved in the metabolism/signaling of hormones, growth, metabolism, and stress responses. WRKY1 [12,21,28] and R2R3MYB [23] are also important proteins that are implicated in the regulation of an array of key biological processes, like primary metabolism, production of secondary metabolites, and defense system.

ZnONP application improved concentrations of K, Fe, and Zn with a strong correlation with target genes. The anatomical study also provides supporting evidence on how ZnONP can improve nutritional status through reinforcement in the vascular system, especially metaxylem tissues. In line with our results, ZnONP at suitable concentration enhanced the concentration of essential nutrients in *Datura Stramonium* [12], *Melissa officinalis* [13], and sorghum [17]. The ZnONP-mediated changes in expression of *ZIP* membrane transporters, hormonal balances, differentiation of vascular system, and source/sink relationship are mechanisms by which ZnONP may influence the homeostasis of nutrients, and consequently metabolism, especially photosynthesis and nitrogen assimilation [20]. A strong correlation among the target genes and nutrients indirectly supports the potential involvements of these genes in the homeostasis of nutrients. For instance, the bHLH transcription factor is a downstream component of auxin that contributes to the establishment of vascular conducting tissues and plant nutrition [39].

The applied ZnONP treatment was associated with an increase in photosynthetic pigments, implying a potential improvement in photosynthesis efficiency and protection (protective roles of carotenoids through xanthophyll cycle); The important mechanisms, thereby improving growth and yield. ZnONP also enhanced nitrate reductase activity and proline concentration, indicating modification in nitrogen primary metabolism. Consistent with these results, ZnONP improved photosynthesis-related traits [14,40], nitrate reductase [15,16], and proline accumulation [12,15,16]. WRKY1 [21], HSFs [25], bHLH [24], R2R3MYB [23] mediate regulation of photosynthesis efficiency in plants. The bHLH transcription factor also serves as a transcriptional positive regulator of proline biosynthesis and nitrogen use efficiency [38].

The observed increase in activities of reactive oxygen species (ROS)-scavenging enzymes and PAL along with an increase in phenolic metabolites suggested the ZnONP-associated induction in the antioxidant system and secondary metabolism. The ZnONP-mediated upregulation in the antioxidant system and secondary metabolites have been well documented [20]. In line with our results, the ZnONP application enhanced the efficacy of the ROS scavenging system through transcriptional upregulation in *POD*, *APX*, *CAT*, and *SOD* genes in rice [41]. Considering the strong positive correlation among the evaluated physiological markers in antioxidant enzymes and secondary metabolism and the target genes, it appears that these transcription factors and MKK2 act as positive upstream regulators of genes involved in the defense system and secondary metabolism. Taking account of knowledge gaps, this study introduces several potential upstream molecular events by which ZnONPs may improve plant metabolism and immunity. A plethora of genes contributed to antioxidants, stress responses, and secondary metabolism are among downstream genes of EREB [9], bHLH [23], R2R3MYB [23], HSFs [25], and WRKY1 [28]. As is well known, up-regulation in *WRKY1* [21,28], *R2R3MYB* [23], *HSFs* [25], *ERFs* [9], and *bHLH* [24] transcription factors confer stress tolerance in plants. Moreover, there is no denying the fact that MAPKs and transcription factors possess critical roles during signaling phenomena. The transcription factors along with MAPKs play substantial determining roles in regulating signaling of phytohormones, activation of stress-related responses, nutrition, differentiation of tissues, and metabolism [27,42]. Besides, it has been stated that upregulation in MAPKs confers stress tolerance [26]. In plants, MKK2 has a close crosslink with MKK1 and MPK4 through which innate immunity is modulated [26]. These molecular findings, therefore, help us to take a clear illustration of how ZnO-NP application can be associated with variation in signaling and transcription of genes and diverse physiological traits.

According to our result, the *HDA3* gene was identified to be a ZnONP-responsive gene, implying the involvement of epigenetic machinery during plant responses to nanoparticles through altering chromatin ultrastructure and gene accessibility. It has been recently reported that ZnONP application mediated changes in expression of *HDA3* in *Datura stramonium* [12] which is consistent with our result. Taking account of other noncompounds, evidence has been recently presented for epigenetic responses to nanoparticles in terms of DNA methylation [27,28]. Upcoming studies are, therefore, necessary to investigate the ZnO-NP-mediated epigenetic changes.

## Conclusion

This study attempted to address the plant molecular and physiological responses to the long-time application of ZnO-NP or its bulk form at low concentration in tomato. The supplements not only modified plant growth, metabolism, productivity, and immunity but also influenced tissue differentiation, especially the vascular system. The ZnO-NP application contributed to up-regulation in the expression of *MKK2* and several important transcription factors, including *bHLH*, *EREB*, *HsfA1a*, *R2R3MYB*, and *WRKY1*. Furthermore, the ZnO-NP treatment mediated downregulation in the *HDA3* gene, manifesting an epigenetic response. Overall, this study provides comprehensive physiological and molecular evidence on how the ZnO-NP application at an appropriate dose may remodel the transcription program, modify primary and secondary metabolism, influence tissue differentiation, and confer stress tolerance in crops. These findings can be useful for filling knowledge gaps and designing future studies on nano-fertilizers or pesticides. This study also underlines the necessity of providing transcriptome and proteome data in future studies.
